# Microbial shifts in dental plaque of children with severe early childhood caries following comprehensive dental treatment under general anesthesia

**DOI:** 10.3389/fcimb.2025.1541785

**Published:** 2025-05-02

**Authors:** Xinyue Wang, Hui Huang, Qizhao Ma, Jing Zou

**Affiliations:** ^1^ State Key Laboratory of Oral Diseases and National Center for Stomatology and National Clinical Research Center for Oral Diseases, West China Hospital of Stomatology, Sichuan University, Chengdu, Sichuan, China; ^2^ Department of Pediatric Dentistry, West China Hospital of Stomatology, Sichuan University, Chengdu, Sichuan, China

**Keywords:** severe early childhood caries, comprehensive dental treatment, high-throughput sequencing, microecological balance, dental plaque

## Abstract

**Objective:**

To elucidate the microbial diversities and compositions of dental plaque in children with severe early childhood caries (S-ECC) before and after comprehensive dental treatment under general anesthesia, contributing evidence for the reestablishment of oral microecological balance.

**Design:**

Twenty children aged 2.2-5.5 years diagnosed with S-ECC without systemic diseases were enrolled in this study. Dental plaque samples were collected before and after treatment, analyzed using high-throughput sequencing on the Illumina Miseq platform. Bioinformatics methods were employed to compare the dental plaque microbial diversity and compositions differences before and after comprehensive dental treatment under general anesthesia.

**Results:**

Significant enhancements in dental plaque microbial diversity were observed post-comprehensive treatment (*P* < 0.05). At the phylum level, the relative abundance of Actinobacteria significantly decreased after treatment (*P* < 0.05). At the genus level, the relative abundance of *Streptococcus*, *Neisseria*, and *Rothia* significantly declined (*P* < 0.05), while *Prevotella* showed a significant increase after treatment (*P* < 0.05).

**Conclusions:**

Following comprehensive dental treatment under general anesthesia, children with S-ECC exhibit significant changes in the microbial diversity and composition of dental plaque, indicating a shift towards a more balanced oral microecological state. This study highlights the importance of dental intervention in positively altering oral microbiota.

## Introduction

Dental caries is the most common chronic infectious oral disease worldwide, particularly affecting young children. It not only affects essential oral functions such as chewing, speech, and aesthetics, but also impacts facial appearance, self-esteem, and psychological well-being (Selwitz et al., 2007). In severe cases, it can exacerbate systemic health problems, such as children growth, cardiovascular disease, immune system disease, and kidney diseases, thereby affecting both oral and overall health in children (Clark and Braun, 2021; Zou et al., 2022). According to the “2022 Global Oral Health Status Report”, dental caries affects 29% of people with permanent teeth (nearly two billion individuals), and has an even more significant presence in primary teeth, with a prevalence rate of 43%, affecting approximately 514 million children (Lemos et al., 2019). Given this high prevalence, the prevention and management of caries in primary dentition remains a global public health priority.

Dental caries is closely associated with an imbalance in the oral microbial ecosystem. According to the ecological plaque hypothesis, diverse and stable oral microbial communities are essential for maintaining oral health (Gao et al., 2018). These balanced communities act as protective barriers against pathogenic shifts in microbial composition (Lamont et al., 2018). This dynamic balance is maintained through intricate interactions among various oral microorganisms and between these microorganisms and host (Blostein et al., 2022). However, alterations in local environmental conditions, such as changes in dietary sugar intake, pH fluctuations, and other biofilm-disrupting factors, can disturb this balance (Li et al., 2005; Rosier et al., 2018). These disturbances favor the growth of acid-producing and acid-tolerant bacteria (Sedghi et al., 2021; Santonocito et al., 2022; Suez et al., 2022). Such shifts reduce microbial diversity and promote the predominance of cariogenic pathogens. This disrupts biofilm stability and contributes to the development of caries (Jakubovics et al., 2021).

Emerging research highlights a marked difference in the oral microbial diversity between caries-free children and those afflicted with early childhood caries (ECC) (Hurley et al., 2019). Notably, a trend towards decreased microbial diversity has been observed in children with ECC, even before the clinical onset of the disease (Becker et al., 2002; Tao et al., 2013). This trend suggests that microbial diversity may serve as an early indicator of caries risk (Valm, 2019; Chen et al., 2020; Xiao et al., 2020). Furthermore, the presence and abundance of specific cariogenic bacteria such as *Streptococcus*, *Actinomyces*, and *Lactobacillus* increase in the context of ECC, while the numbers of health-associated bacterial species, such as those belonging to the *Bacteroidaceae* family, are significantly reduced (Xiao et al., 2019; Garcia et al., 2021; Li et al., 2023).

Severe early childhood caries (S-ECC), represents an extreme manifestation of ECC, defined as smooth surface caries in children younger than 36 months old or decayed missed filled surfaces (dmfs) ≥ 4 (3 years old), dmfs ≥ 5 (4 years old) and dmfs ≥ 6 (5 years old) (Zou et al., 2022). Children with S-ECC are very young and often experience anxiety and fear towards dental treatment (Seligman et al., 2017). It has been reported that 3%-43% of these children are unable to cooperate or even refuse treatment due to anxiety which delays their treatment time (Folayan et al., 2004). Due to the physiological and psychological characteristics of children, in order to quickly restore the function of the affected teeth, children with S-ECC who have complex treatment plans are often treated with one-time comprehensive dental treatment under general anesthesia in clinical practice to restore the shape of the teeth and their masticatory function in the shortest time (Schmoeckel et al., 2020; Kries et al., 2023). Despite the advancements in treatment modalities and our growing understanding of the microbial etiology of ECC, there remains a significant gap in our knowledge regarding the impact of comprehensive dental treatments on the oral microbial communities of children with S-ECC. Specifically, the changes in the diversity and composition of dental plaque biofilms following such treatments have not been explored.

This study explores the alterations in dental plaque microbial communities of children with diagnosed with S-ECC, both before and after comprehensive treatment under general anesthesia. By investigating the shifts in microbial diversity and composition associated with treatment, this research aims to provide new insights into the microbial basis for comprehensive dental interventions reestablishing oral microecological balance.

## Materials and methods

### Study subjects selection

Twenty children aged 2.2-5.5 years with dental caries were selected from the Department of Pediatric Dentistry, West China Hospital of Stomatology, Sichuan University, Chengdu, Sichuan, China, based on the following criteria:

Inclusion criteria:

1) Normal mental and intellectual abilities, with a Body Mass Index (BMI) within the normal range.
2) Clinically diagnosed with S-ECC.
3) Eligible for treatment under general anesthesia with parental consent for the treatment plan.

Exclusion criteria:

1) Presence of serious systemic diseases or infectious diseases, for which pediatricians do not recommend surgery under general anesthesia.
2) Use of antibiotics within three months prior to sampling, or a history of systemic medication use.
3) Prior dental disease treatments.
4) Dental structure abnormalities, such as enamel hypoplasia or dentinogenesis imperfecta.

All subjects and their guardians voluntarily participated in the study, with informed consent forms signed by the guardians.

### Collection of plaque samples

Parents were explicitly instructed that the children should refrain from brushing their teeth starting the night before sampling and continue avoiding tooth brushing on the morning of the plaque collection. Plaque samples were collected consistently from standardized locations during both preoperative assessments and the one-month postoperative follow-up. Specifically, plaque samples were collected exclusively from supragingival plaque deposits on the buccal enamel surfaces of both the first and second primary molars on the left and right sides of the mandible. Samples were carefully scraped using sterile oral swabs under standardized conditions, ensuring only supragingival plaque was included and avoiding contamination with gingival crevicular fluid, gingival tissue. Following comprehensive dental treatment under general anesthesia, parents received detailed oral hygiene instructions, including proper tooth brushing techniques, dietary recommendations, and regular oral hygiene practices to maintain oral health and minimize caries recurrence. The collected plaque samples were immediately placed in 1 mL TE buffer (pH value 7.4) and transported to the laboratory within 2 hours on ice, and stored at -20°C for further DNA extraction.

### DNA extraction and 16S rRNA gene sequencing

Total bacterial DNA was extracted using the QIAamp^®^ DNA Micro Kit (QIAGEN, USA), following the manufacturer’s instructions. The extracted plaque sample DNA was stored at -80°C and sent to Shanghai Meiji Biomedical Technology Co., Ltd., under dry ice refrigeration conditions for subsequent sequencing and bioinformatic analysis. PCR amplification targeting the V3-V4 region of the bacterial 16S rRNA gene was performed, with the products analyzed using the Illumina Miseq sequencing platform.

### Microbial diversity analysis

The Usearch software (version 7.1) with the RDP classifier Bayesian algorithm was used to perform taxonomic analysis on OTU representative sequences at 97% similarity level, and the community composition of each sample was counted at various levels (domain, kingdom, phylum, class, order, family, genus, species). The richness and diversity of the microbial communities in the samples were analyzed using Chao, ACE, Shannon, and Simpson indexes.

### Microbial community difference analysis

The indexes of microbial diversity in dental plaque and the relative abundance of various bacterial communities before and after general anesthesia treatment in children were analyzed for significant differences using SPSS 18.0 software and paired t-test. The results were expressed as mean ± standard deviation, with a significant level of α=0.05.

## Results

### Clustering analysis of operational taxonomic units

Through Illumina MiSeq sequencing, a total of 1,782,512 sequences were obtained from 40 samples, with 802,183 sequences before treatment, averaging 40,109 sequences per sample, and 980,329 sequences after treatment, averaging 49,016 sequences per sample. The average length of each sequence was 442.98 bp, with nearly all sequences falling within the ranges of 421-440 bp (27.11%) and 441-460 bp (72.87%), suitable for further analysis (**Table 1**).

**Table 1:** Basic sequencing information of supragingival plaque samples collected from 20 children with severe early childhood caries (S-ECC) before and one month after comprehensive dental treatment under general anesthesia.

	Sample number	Total sequence number	Total base number (bp)	Average length (bp)
Optimized data	40	1,782,512	789,614,496	442.98

Based on the principle of similarity greater than 97%, the obtained high-quality sequences were compared with the SILVA database (Quast et al., 2013), and a total of 259 OTUs were obtained, with 251 before treatment and 252 after treatment, sharing 244 OTUs between the two groups (Fouts et al., 2012). The average OTU count for plaque microbiome was 142 ± 29 before treatment, significantly increasing to157 ± 20 after treatment (*P* < 0.05).

### OTU-based analysis of microbial diversity

After undergoing comprehensive treatment of dental disease under general anesthesia, Chao1 value and Shannon index significantly increased (*P* < 0.05), while there were no significant differences in the ACE value and Simpson index (*P* > 0.05), suggesting an increase in the microbial diversity of plaque biofilms. The coverage values before and after treatment exceeded 0.999, indicating high library coverage and the sequencing results accurately represented the microbial situation in the samples (**Table 2**).

**Table 2:** Alpha diversity analysis of supragingival plaque microbial communities before and after treatment.

Time	ACE	Chao1	Shannon	Simpson	Coverage
Before treatment	155 ± 27.7	156 ± 27.9	3.16 ± 0.48	0.0990 ± 0.08	0.999
After treatment	167 ± 21.8	171 ± 23.5	3.41 ± 0.39	0.0783 ± 0.05	0.999
*P*-value	0.050	0.023^*^	0.039^*^	0.155	

*P < 0.05.

### Composition analysis of microbial communities

**Figure 1:**
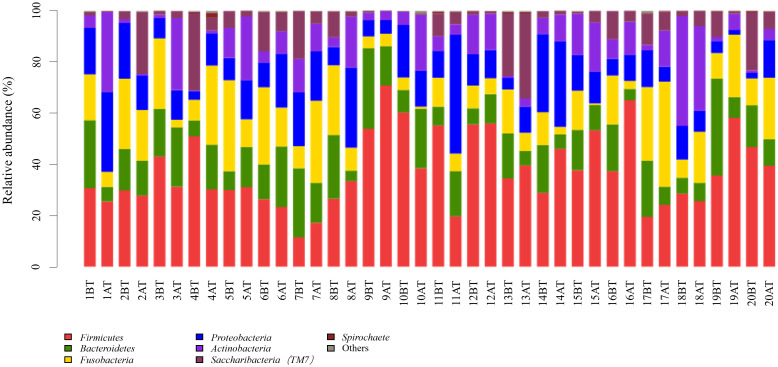
Relative abundance of bacterial phyla in supragingival plaque samples collected from 20 children diagnosed with S-ECC before and one month after comprehensive dental treatment under general anesthesia. Each bar represents the phylum-level taxonomic composition of a sample, calculated based on the percentage of total sequences. BT, before treatment; AT, after treatment.

**Figure 2:**
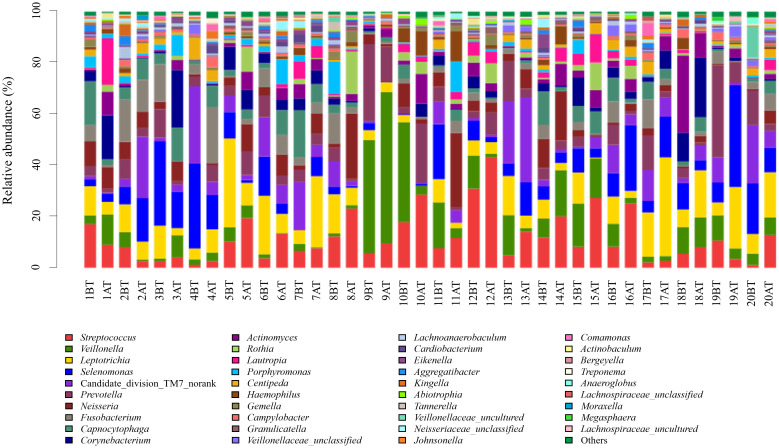
Relative abundance of bacterial genera in supragingival plaque samples collected from 20 children diagnosed with S-ECC before and one month after comprehensive dental treatment under general anesthesia. Each bar represents the genus-level taxonomic composition of a sample, calculated based on the percentage of total sequences. BT, before treatment; AT, after treatment.

The 259 OTUs were classified into 12 phyla, 20 classes, 30 orders, 46 families, and 87 genera. At the phylum level, 12 phylum bacteria were detected (**Figure 1**), with more than 99% primarily belonging to six dominant phyla: Firmicutes (37.5%), Fusobacteria (15.1%), Bacteroidetes (14.8%), Proteobacteria (14.2%), Actinobacteria (10.6%), and Saccharibacteria (TM7) (7.5%). At the genus level, bacteria from 87 genera were detected between the two groups (**Figure 2**), with genera having a relative abundance >1% including *Actinobacillus* (3.8%), *Corynebacterium* (4.3%), *Rotella* (2.1%), and *Porphyromonas* (2.1%). *Prevotella* (7.2%), *Capnocytophaga* (4.7%), *Streptococcus* (11.6%), *Centipeda* (1.7%), *Selenomonas* (9.6%), *Veillonella* (9.3%), *Fusobacterium* (4.6%), *Leptotrichia* (10.5%), *Lautropia* (2.3%), *Neisseria* (5.6%), *Campylobacter* (1.0%), *Haemophilus* (1.7%), and Candidate_division_TM7_norank (7.5%).

### Analysis of phylum-level differences in microbial composition

**Figure 3:**
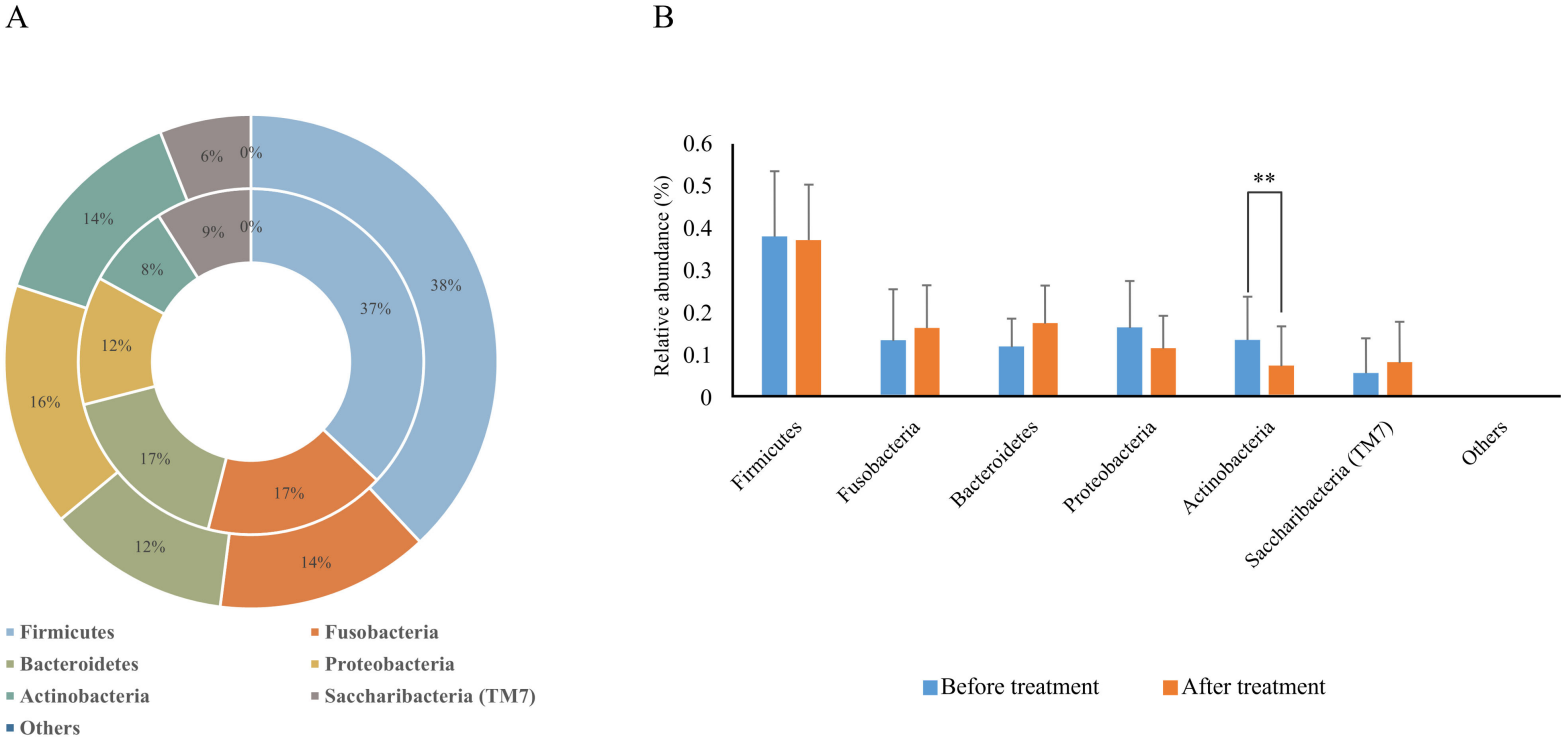
Relative abundance of dominant bacterial phyla in supragingival plaque samples collected from 20 children diagnosed with S-ECC before and one month after comprehensive dental treatment under general anesthesia. **(A)** Taxonomic composition at the phylum level is shown. The outer circle represents before-treatment samples and the inner circle represents after-treatment samples. **(B)** Bar plots of mean relative abundance (%) of each dominant phylum. **P < 0.01.

Before treatment, the relative abundance of dominant phyla in plaque biofilms was Firmicutes (37.9%), Proteobacteria (16.4%), Actinobacteria (13.7%), Fusobacteria (13.6%), Bacteroidetes (12.2%), and Saccharibacteria (TM7) (5.9%). After treatment, the relative abundance was Firmicutes (37.1%), Bacteroidetes (17.4%), Clostridium (16.8%), Proteobacteria (11.9%), Saccharibacteria (TM7) (9.0%), and Actinobacteria (7.5%), with Actinobacteria being significantly decreased compared to before treatment (**Figure 3**, **Table 3**).

**Table 3:** Comparison of relative abundance of bacterial phyla in supragingival plaque before and after treatment.

Time	Firmicutes	Fusobacteria	Bacteroidetes	Proteobacteria	Actinobacteria	Saccharibacteria (TM7)
Before treatment	37.9 ± 15.5	13.6 ± 11.9	156 ± 27.9	16.4 ± 11.1	13.7 ± 10.2	5.9 ± 8.3
After treatment	37.1 ± 13.1	16.8 ± 9.6	17.4 ± 8.9	11.9 ± 7.5	7.5 ± 9.6	9.0 ± 8.9
*P*-value	0.831	0.359	0.081	0.085	0.008^**^	0.156

**P < 0.01.

### Analysis of genus-level differences in microbial composition

**Figure 4:**
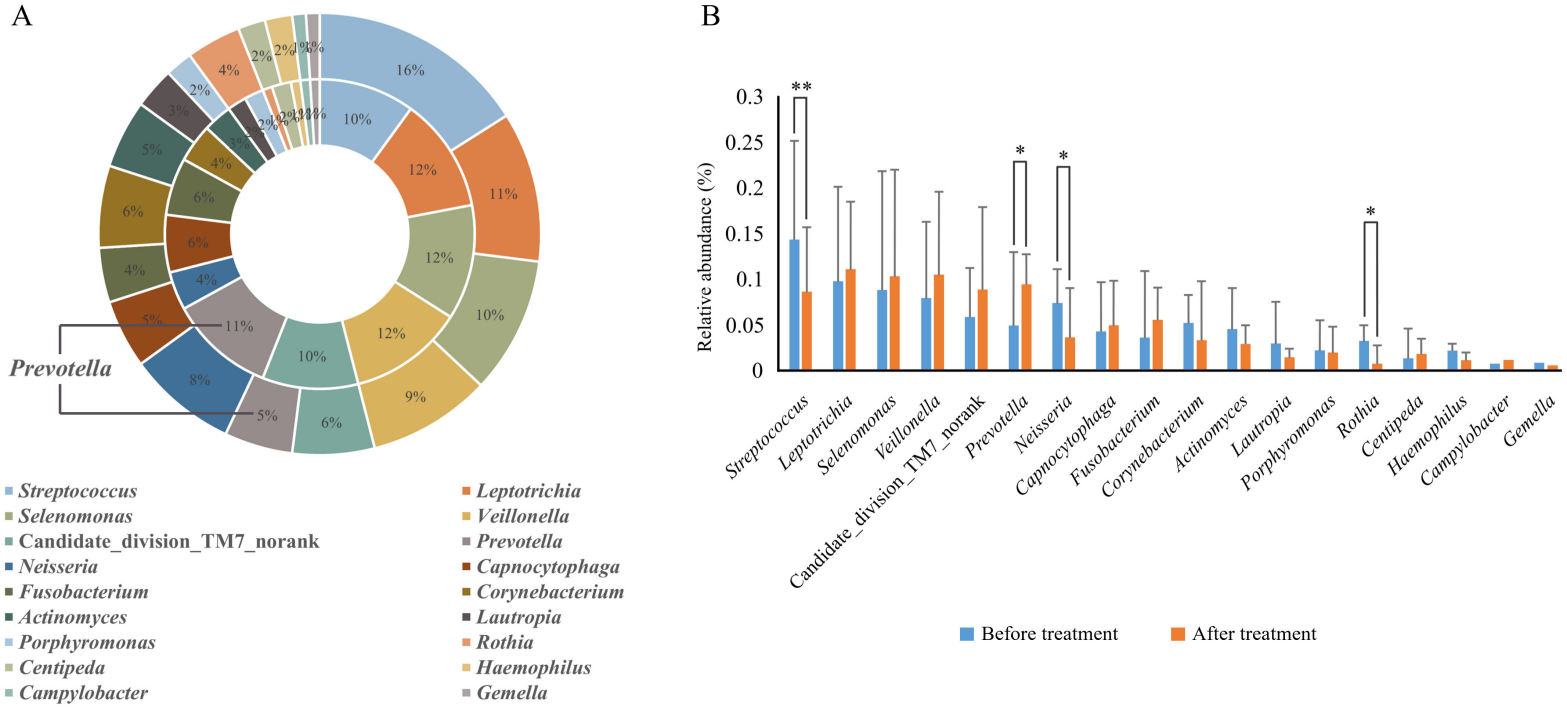
Relative abundance of dominant bacterial genera (> 1% in average relative abundance) in supragingival plaque samples collected from 20 children diagnosed with S-ECC before and one month after comprehensive dental treatment under general anesthesia. **(A)** Taxonomic composition at the genus level is shown. The outer circle represents before-treatment samples and the inner circle represents after-treatment samples. **(B)** Bar plots of mean relative abundance (%) of each dominant genus. *P < 0.05 or **P < 0.01.

Before treatment, 85 genera were detected, with those having a relative abundance > 1% being, in descending order, *Streptococcus* (14.5%), *Leptothrix* (9.8%), *Lunomonas* (8.9%), *Veillonella* (8.0%) and *Neisseria* (7.5%), Candidate_division_TM7_norank (5.9%), *Corynebacterium* (5.2%), *Prevotella* (5.0%), *Actinobacillus* (4.7%), *Fibrophagocytophages* (4.4%), *Fusobacterium* (3.7%), *Roxella* (3.4%), *Lautropia* (3.1%), *Porphyromonas* (2.2%), *Haemophilus* (2.2%), and *Centipeda* (1.4%); After treatment, 86 genera were detected, with the relative abundance > 1% being *Leptothrix* (11.1%), *Veillonella* (10.7%), *Lunomonas* (10.3%), *Prevotella* (9.5%), Candidate_division_TM7_norank (9.0%), *Streptococcus* (8.8%), *Clostridium* (5.6%), *Capnophage* (5.1%), *Neisseria* (3.8%), *Corynebacterium* (3.3%), *Actinobacillus* (3.0%), *Porphyromonas* (2.0%), *Centipeda* (2.0%), *Lautropia* (1.5%), *Campylobacter* (1.2%), and *Hemophilus* (1.2%). Post-comprehensive treatment, four genera with a relative abundance > 1% showed significant changes (*P* < 0.05). The relative abundance of *Streptococcus* decreased from 14.5% before treatment to 8.8% after, *Neisseria* from 7.5% to 3.8%, *Rothia* from 3.4% to 0.8%, while *Prevotella* increased from 5.0% to 9.5%. No significant differences were observed in the relative abundance of other bacteria genera before and after treatment (*P* > 0.05) (**Figure 4**, **Table 4**).

**Table 4:** Comparison of relative abundance of bacterial genera (> 1% in average relative abundance) in supragingival plaque before and after treatment.

Bacterial genus	Average	Before treatment	After treatment	*P*-value
*Streptococcus*	11.6	14.5 ± 10.8	8.8 ± 7.0	0.003^**^
*Leptotrichia*	10.5	9.8 ± 10.3	11.1 ± 7.5	0.659
*Selenomonas*	9.6	8.9 ± 10.1	10.3 ± 9.6	0.640
*Veillonella*	9.3	8.0 ± 13.0	10.7 ± 11.8	0.287
Candidate_division_TM7_norank	7.5	5.9 ± 8.3	9.0 ± 8.9	0.156
*Prevotella*	7.2	5.0 ± 5.5	9.5 ± 9.0	0.049^*^
*Neisseria*	5.6	7.5 ± 8.0	3.8 ± 3.2	0.042^*^
*Capnocytophaga*	4.7	4.4 ± 3.7	5.1 ± 5.4	0.659
*Fusobacterium*	4.6	3.7 ± 5.3	5.6 ± 4.8	0.225
*Corynebacterium*	4.3	5.2 ± 7.3	3.3 ± 3.5	0.230
*Actinomyces*	3.8	4.7 ± 3.2	3.0 ± 6.5	0.213
*Lautropia*	2.3	3.1 ± 4.5	1.5 ± 2.0	0.114
*Rothia*	2.1	3.4 ± 4.6	0.8 ± 1.0	0.012^*^
*Porphyromonas*	2.1	2.2 ± 3.4	2.0 ± 2.8	0.872
*Centipeda*	1.7	1.4 ± 1.7	2.0 ± 2.1	0.231
*Haemophilus*	1.7	2.2 ± 3.3	1.2 ± 1.5	0.147
*Campylobacter*	1.0	0.7 ± 0.9	1.2 ± 0.9	0.107

*P < 0.05 or **P < 0.01.

## Discussion

ECC, particularly S-ECC, is a biofilm-mediated condition that poses significant challenges in pediatric dentistry. S-ECC can lead to severe complications in primary dentition, such as pulp and periapical diseases, potentially affecting the development of permanent teeth. Comprehensive dental treatment under general anesthesia offers an effective solution for S-ECC by removing carious lesions in a single session, thus addressing both the immediate dental needs and anxiety-related challenges faced by young patients. However, reports on the microbial community shifts within dental plaque biofilms pre- and post-comprehensive treatment under general anesthesia in children with S-ECC are scarce.

Our study employed metagenomics to analyze the microbial dynamics of dental plaque in children with S-ECC, pre- and post-comprehensive treatment under general anesthesia. Post-comprehensive treatment, we observed a significant increase in microbial diversity, as evidenced by increases in the Chao1, and Shannon index, aligning with previous findings by Li et al. (2007) and Tao et al. (2013). This enrichment in microbial diversity post-comprehensive treatment mirrors the microecological equilibrium found in caries-free children, suggesting that comprehensive treatment positively influences oral microbial ecology.

Our results suggest that the relative abundance of the Actinobacteria phylum significantly decreases post-comprehensive treatment, implying a potential inverse correlation with oral health, consistent with Tang et al.’s findings (Tang et al., 2003). *Actinomyces* and *Streptococcus* have been shown to exhibit synergistic interactions in the early stages of carious development (Sarkonen et al., 2000), where the cariogenic role of Actinobacteria is to promote caries by altering the microecological balance. At the genus level, post-comprehensive treatment analysis indicated a significant reduction in the relative abundance of genera *Streptococcus*, *Rothia*, and *Neisseria*, which may be positively correlated with the progression of caries. *Streptococcus mutans* and *Streptococcus sobrinus* within the *Streptococcus* genus are recognized as primary etiological agents of S-ECC (Veena and Nagarathna, 2020), with a higher caries rate in children harboring both species compared to those with only *S. mutans* (Zhou et al., 2011). *Rothia* species’ prevalence and activity within carious lesions (Belda-Ferre et al., 2015), and their association with dentine caries and subgingival biofilm formation in anaerobic conditions (Jagathrakshakan et al., 2015), support their relevance in caries pathology. The decline in *Neisseria* post-comprehensive treatment suggests a potential role in S-ECC’s pathogenesis, warranting further investigation.

Additionally, the relative abundance of *Prevotella* significantly increased post-comprehensive treatment in this study. While *Prevotella* is a common and abundant genus in the oral cavity, its role in oral health and disease remains complex and species-dependent. Previous studies have indicated that elevated levels of *Prevotella* in individuals with active caries, suggesting that certain species within this genus may contribute to dysbiosis and disease progression (Kanasi et al., 2010; Tanner et al., 2011). However, other *Prevotella* species, such as *Prevotella melaninogenica* and *Prevotella histicola*, are frequently found in the healthy oral microbiome and may contribute to maintaining ecological balance (Jiang et al., 2016; He et al., 2018). Therefore, the increased abundance of *Prevotella* in our study may not directly imply a heightened risk of caries recurrence, but could reflect a shift toward greater microbial diversity and ecological restructuring post-comprehensive treatment. It is also important to consider that this change may be influenced by inter-individual variability in factors such as diet, oral hygiene, and lifestyle, as well as the relatively small sample size of the present study. Further species-level and functional analyses are needed to determine whether the increase in *Prevotella* represents a beneficial reestablishment of microbial homeostasis or serves as an early microbial indicator of caries risk.

The research conducted by Klinke et al. (2014) indicates that while the abundance of cariogenic bacteria significantly decreases following caries treatment, this reduction may be transient, with the potential for these taxa to rebound over time. In our study, we observed significant shifts in the microbial composition one month after comprehensive dental treatment under general anesthesia, suggesting an initial positive response in terms of microbial diversity. However, due to the dynamic nature of oral microbial communities, influenced continuously by environmental factors, dietary habits, and oral hygiene practices, the long-term stability and durability of these treatment-related microbial shifts remain unclear and warrant further investigation. Additionally, the interpretation of our findings is constrained by several limitations, including a small sample size, potentially compromising statistical power and the generalizability of the results. Furthermore, our sequencing approach was limited to genus-level identification, thus precluding a deeper understanding of species-specific shifts that might provide clearer insights into cariogenic or health-related microbial dynamics. Future research incorporating larger cohorts, extended follow-up periods (e.g., 3 to 6 months or longer), and species-level metagenomic analyses will be necessary to better elucidate the sustained effects of comprehensive dental treatment on oral microbial homeostasis and the clinical relevance of these microbial changes.

In conclusion, significant changes in microbial diversity and composition are observed in the dental plaque biofilms of children with S-ECC following comprehensive dental treatment under general anesthesia. The increase in microbial diversity and the concomitant reduction in cariogenic microorganisms of post-comprehensive treatment indicate a shift toward a healthier, more balanced, and stable microbial state. These findings highlight the potential of comprehensive dental treatment in promoting a favorable shift in oral microbiota, emphasizing its theoretical and practical implications in the management of S-ECC from a microbiological perspective.

## Data Availability

The original contributions presented in the study are included in the article/supplementary material. Further inquiries can be directed to the corresponding authors.
